# Anatomical and Physiological Differences between the Caucasian and African Races

**Published:** 1881-06

**Authors:** Harvey L. Byrd

**Affiliations:** Professor of Obstetrics in Washington University of Baltimore; former Professor of Materia Medica and Therapeutics in Savannah Medical College; and late Professor of Principles and Practice of Medicine in Oglethorpe Medical College of Georgia, etc.


### POPULAR SCIENCE DEPARTMENT.

### ANATOMICAL AND PHYSIOLOGICAL, DIFFERENCES BETWEEN THE CAUCASIAN AND AFRICAN RACES.

BY HARVEY L. BYRD, M. D.

Professor of Obstetrics in Washington University of Baltimore; former Professor of Materia Medica and Therapeutics in Savannah Medical College; and late Professor of Principles and Practice of Medicine in Oglethorpe Medical College of Georgia, etc.

In a recent lecture, Professor Agassiz remarked: "I have pointed out over a hundred specific differences between the bonal and nervous systems of the white man and the negro. Indeed, their forms are alike in no particular. There is not a bone in the negro's body that is relatively the same shape, size, articulation, or chemically of the same composition, as that of the white man. The negro's bones contain a far greater percentage of calcareous salts than those of the white man; even the negro's blood is chemically a very different fluid from that which courses in the veins of the white man. The whole physical organism of the negro differs quite as much from the white man's as it does from that of the chimpanzee—that is, in his bones, muscles, nerves and fibers, the chimpanzee has not much further to progress to become a negro, than a negro has to become a white man. This fact science inexorably demonstrates. * * * * Climate has no more to do with the difference between the white man and the negro than it has with that between the negro and the chimpanzee; or than it has between the horse and the ass; or the eagle and the owl. Each is a distinct and separate creation. The negro and the white man were created as different as the owl and the eagle. They were designed to fill different places in the system of nature. The negro is no more a negro by accident or misfortune, than the owl is the kind of bird he is by accident or misfortune. The negro is no more the white man's brother, than the owl is the sister of the eagle; or the ass is the brother of the horse. How stupendous, and yet how simple is the doctrine, that the Almighty Maker of the Universe has created inherent species of the lower animals, to fill the different places and offices in the grand scenery of nature." ("Medical and Surgical Reporter," vol. 16, page 453). The following skeleton facts, developed during an experience of nearly a quarter of a century, as a physician, in the rice growing region of South Carolina and Georgia, is subjoined for the purpose of presenting a few prominent and highly important physiological and therapeutical differences which exists between the Caucasian and African races. The negro is far less liable to malarial fevers in the rice swamps of the South than the white man under the same circumstances. Instances so frequently repeated as to leave no doubt of the constant operation of the same laws in their production and development, have come under the observation of the writer, confirmatory of this fact, that it would alone go very far toward the establishment, in his mind, of the diversity of origin of the two races, even were other evidences wanting to do so. It was as well known, a few years ago, to the negro as to overseer, or proprietor of the rice plantations on the Pee Dee, Waccamaw, Santee and other rivers in the rice regions of South Carolina, that the former could lie down and sleep upon the ground in the open night air, even when heated by the fatigues incident to the rice harvest, with perfect immunity, or exemption from miasmatic fever: and that a nap of half an hour, while sitting in the barnyard superintending the night duties of the harvest, entailed, with almost unfailing certainty, an attack of fever, within *nine* days, on the overseer, or any other white person who might be so unfortunate as to be overtaken by sleep when so exposed. This occurred, too, where the negro and the white man were natives or long residents of the locality. So constant and universally was this the case, that great effort was made by white men to keep awake while superintending the night duties incident to harvest season. Yet they could sleep in their residences contiguous to the plantations with perfect impunity, for hours, *during the day.*

From June to November, the malarial period, the whites found it necessary to remove from the plantations sufficiently far to place a forest of pine or other large growth between their temporary retreats and the rice swamps, as a matter of *self-preservation*, or, at least, protection against bilious, remittent and intermittent fevers; while the negroes remained, during their absence, healthy and happy, increasing and multiplying, as well during the summer and autumn as at any other periods or seasons of the year. The native African enjoyed equal immunity from miasmatic fever with the negro born on the soil. On the visitation of cholera; during the residence of the whites on the plantations, the negro alone was the sufferer, and this, too, whilst the former was humanely and carefully ministering to his wants and necessities. It is due alike to science and to truth to state, that the negroes, on all the plantations that came under my professional care, were well fed on good and wholesome food, properly clad and shod, not over-worked, and provided with comfortable houses. Again, the negro was almost exempt, during the yellow fever epidemics which proved in so many instances so devastating to the white population in certain cities in the South. His exemption from this disease is often most strikingly manifest during an epidemical visitation. One of the most pleasing episodes in my professional experience in yellow fever, was the kindness and care manifested by the negro for the suffering and dying whites, during an epidemic of that disease. From his almost complete exemption from it, and his naturally kind and gentle disposition toward the superior race, he was wont to constitute, in many cases, the sole attendant and nurse for the sick, as well as undertaker and sexton for the dead. The fidelity and kindness thus manifested by him is worthy of the highest praise. Amid thousands of yellow fever patients that received professional attention at my hands, a case of *black vomit* was never seen in a negro. Nor were even the few cases, where the general phenomena of yellow fever presented themselves in the African of full blood, difficult of management or marked by symptoms of more than ordinary gravity. Mulattoes were more liable to be attacked than negroes, and less liable than white persons. The susceptibility is—according to my observation and experience—in direct ratio or relation to the larger proportion of white blood in *mix-blooded* persons, during the prevalence of an epidemic of yellow fever. The comparative susceptibility of the white and negro races to several other diseases might be mentioned, and interesting parallel and differential phenomena adduced, did our subject seem to require it for further illustration or support. We observe, however, in passing, that there are many well defined and constant phenomena, which, considered physiologically and therapeutically, are not only of great value in a large number of diseases, but which go very conclusively to prove, to the philosophical observer, that *great natural and original differences and distinctions* exist between the African and Caucasian races. Thus,

*cæteris paribus*, an epidemical visitation effecting simultaneously both races, will evince phenomena in the white race of a *sthenic*, and in the negro a more or less *asthenic*, condition will be found. The treatment as well as general symptoms establishing clearly these important facts. In some such seasons, for example, antiphlogistics, and even venesection, has been found the remedy, *par excellence*, in the white race; whilst depletives and arterial sedations were obviously detrimental, and sustaining or even stimulating remedies loudly called for in the African, often in the early stages of disease. These facts are the more important and significant when we remember that such general treatment would have to be reversed, in managing an epidemic among the laboring and the more comfortably circumstanced classes of the *white race.*

Again, the negro in health habitually consumes less oxygen during respiration than the white man. Intelligent physicians should turn this fact to useful account in the professional management of their diseases. Among other evidences of this fact, may be mentioned the constant tendency of the negro to *cover his head and face* when going to sleep,particularly in cool, and even moderately warm weather. Nothingwas more common during winters of the South than to find him at night with vertex to the fire, and the blanket or other covering drawn closely over the head and face, while the feet and shins were naked and exposed to the cold, and in this condition sleeping soundly andpleasantly.

On a professional visit, late of a winter's night, half a dozen, and occasionally more, might have been seen with closely covered heads and faces, and uncovered feet and legs. The head to the fire-place and the feet projecting into the room—resembling the radii of a semicircle—and that, too, when they were supplied with comfortable sleeping apartments and had sufficient bed covering in another part of the house.

The negro seems to require more carbon for the well being of his organism, and far less oxygen, at least during sleep, than the white man. This fact may be seen in the *universal tendency to cover the face and head*, alluded to above, even in moderately warm weather; and not a few instances have been observed where the face and head were covered before going to sleep, even in midsummer. For many years I have successfully treated the negro quite differently from the course pursued in managing the same disease in the white man, under even almost precisely similar circumstances. He bears full doses of opium and its preparations, for example, less favorably than the white man, and cannot be depleted to the same extent without injury—requiring an earlier resort to stimulants and supporting treatment than the superior race, in the same disease and in precisely similar surroundings. Instances might be multiplied to any desirable extent, and differentialism instituted, to show that while a sufficient number of *symptoms* may occur to enable us to determine the existence of certain diseases common to both races, a large number and even important *signs* may be found that are *peculiar* to each. The inability of the African to withstand the effects of considerably reduced temperature, is well known, and the comfort he derives from hot weather might, if time permitted, be brought out in striking comparison with the white man, and found an additional evidence of the *original diversity* of the races. Nearly three hundred years of residence, association and contact, on this continent, with the white man, have wrought no change in his physical and physiological characteristics; and the inadequacy of *time and circumstances to effect any essential* or even appreciable alteration in his anatomical structure, at least, is wholly set at rest by the monumental revelations of Egypt. The foregoing and other facts which might be adduced, force the conviction upon our mind, that he was not only cast *originally* in form and color in which he appears to us now, but that he had an existence in the jungles of his native Africa, long ages ere the white man was created in the image of his Maker, or permitted to enjoy the beauty and bliss of an Oriental Paradise.

NOTE.—The above article was published in the *Baltimore Medical Journal* in 1870, and is reproduced here at the particular request of several of the subscribers to THE INDEPENDENT PRACTITIONER. H. L. B.

